# Supplementary figures and images for: Deforestation and human proximity influence Trypanosoma cruzi infection in palm-dwelling triatomines
Source: PLoS One. 2026 May 18;21(5):e0349311. doi: 10.1371/journal.pone.0349311 (PMC13183234; doi:10.1371/journal.pone.0349311)

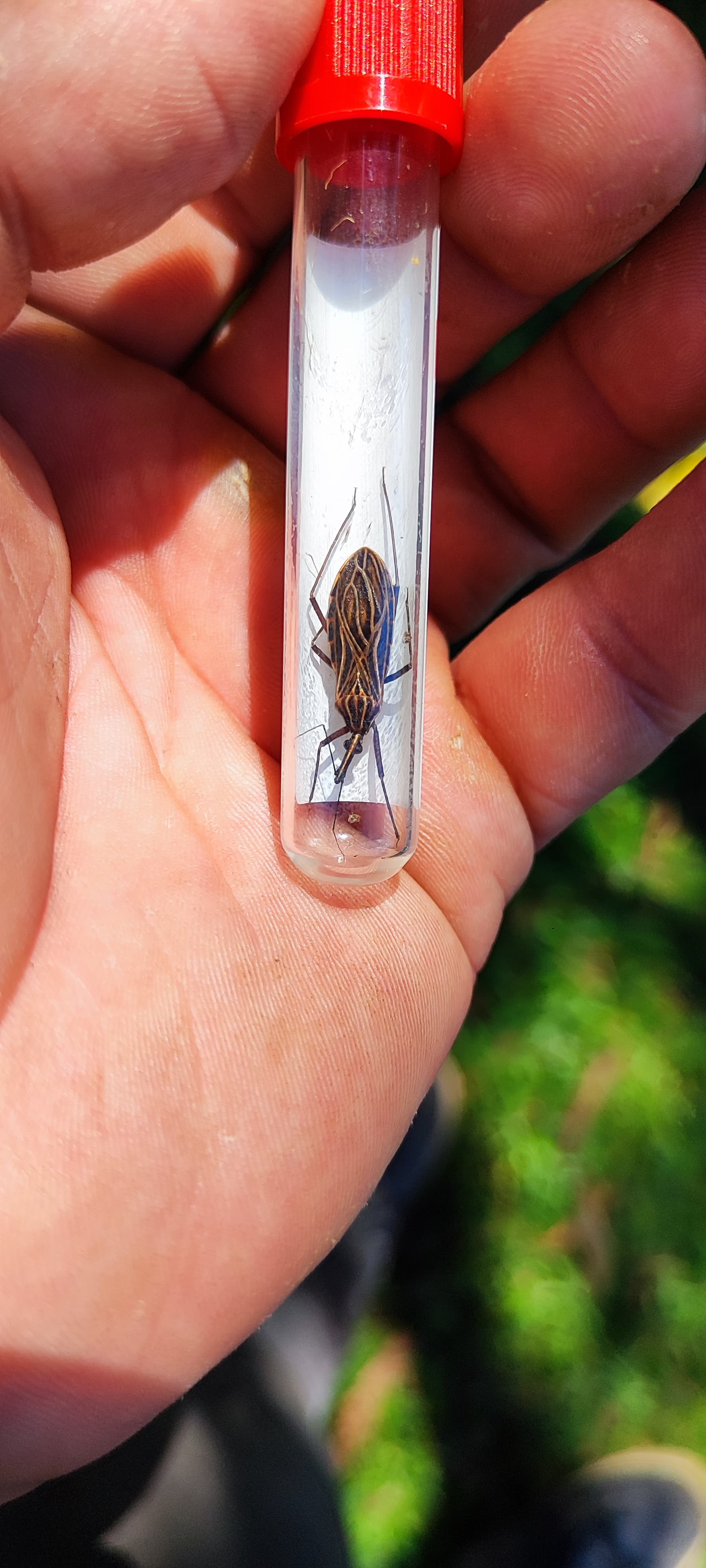

Supplement: S1 Fig — (JPEG) [file pone.0349311.s001.jpeg]
