# Supplementary material for: Deforestation and human proximity influence Trypanosoma cruzi infection in palm-dwelling triatomines
Source: PLoS One. 2026 May 18;21(5):e0349311. doi: 10.1371/journal.pone.0349311 (PMC13183234; doi:10.1371/journal.pone.0349311)
Supplement: S1 Table — (DOCX) [file pone.0349311.s002.docx]

**S1 Table. Molecular detection of *T*. *cruzi* and *T*. *rangeli* and identification of blood meal sources in *Rhodnius* spp. collected from palm trees across landscape sites in Cruzeiro do Sul, Acre state, Brazil, in 2022 and 2024.**

| **Site^a,b^** | **Specimen** | **Year** | **Triatomine** | **Biological sample** | **Sat qPCR** | **PCR 18S** | **Sequencing 18S** | **GenBank ID** | **DTU** | **cytb** |
| --- | --- | --- | --- | --- | --- | --- | --- | --- | --- | --- |
| A4 | 1 | 2022 | *Rhodnius* | Nymph | — | — | — | — | — | — |
| A4 | 2 | 2022 | *Rhodnius* | Nymph | — | — | — | — | — | *Copeoglossum nigropunctatum* |
| A4 | 3 | 2022 | *Rhodnius* | Nymph | — | Positive | *T. rangeli* | PQ846621 | — | *Phylander opossum* |
| A4 | 4 | 2022 | *Rhodnius* | Nymph | *T. cruzi* | Positive | *T. cruzi* | PQ846620 | TcI | — |
| A4 | 5 | 2022 | *Rhodnius* | Nymph | — | — | — | — | — | — |
| A4 | 6 | 2022 | *Rhodnius* | Nymph | — | — | — | — | — | — |
| A4 | 7 | 2022 | *Rhodnius* | Nymph | — | — | — | — | — | — |
| A4 | 8 | 2022 | *Rhodnius* | Nymph | — | — | — | — | — | — |
| A4 | 9 | 2022 | *Rhodnius* | Nymph | — | — | — | — | — | — |
| A4 | 10 | 2022 | *Rhodnius* | Adult male | *T. cruzi* | Positive | *T. cruzi* | PQ846623 | TcI | — |
| A5 | 1 | 2022 | *Rhodnius* | Nymph 1 | — | — | — | — | — | — |
| A7 | 1 | 2022 | *Rhodnius* | Adult female | *T. cruzi* | Positive | *T. cruzi* | PQ846616 | TcI | *Didelphis marsupialis* |
| A7 | 2 | 2022 | *Rhodnius* | Nymph 3 | *T. cruzi* | Positive | *T. cruzi* | PQ846617 | TcI | — |
| A7 | 3 | 2022 | *Rhodnius* | Nymph 4 | *T. cruzi* | Positive | *T. cruzi* | PQ846618 | TcI | — |
| A7 | 4 | 2022 | *Rhodnius* | Nymph 3 | *T. cruzi* | Positive | *T. cruzi* | PQ846619 | TcI | — |
| A7 | 5 | 2022 | *Rhodnius* | Nymph 5 | *T. cruzi* | Positive | *T. cruzi* | PQ846622 | TcI | — |
| A19 | 1 | 2022 | *Rhodnius* | Adult female | *T. cruzi* | Positive | *T. cruzi* | PQ846625 | TcI | — |
| A20 | 1 | 2022 | *Rhodnius* | Nymph 1 | — | — | — | — | — | — |
| A20 | 2 | 2022 | *Rhodnius* | Nymph 1 | — | — | — | — | — | — |
| A20 | 3 | 2022 | *Rhodnius* | Nymph 1 | — | — | — | — | — | — |
| A20 | 4 | 2022 | *Rhodnius* | Nymph 2 | — | — | — | — | — | — |
| A20 | 5 | 2022 | *Rhodnius* | Nymph 3 | — | — | — | — | — | *Copeoglossum nigropunctatum* |
| A20 | 6 | 2022 | *Rhodnius* | Adult female | — | — | — | — | — | — |
| A3 | 1 | 2024 | *Rhodnius* | Nymph 4 | — | — | — | — | — | *Homo sapiens* |
| A4 | 1 | 2024 | *Rhodnius* | Nymph 3 | — | — | — | — | — | — |
| A5 | 1 | 2024 | *Rhodnius* | Nymph 1 | — | — | — | — | — | — |
| A5 | 2 | 2024 | *Rhodnius* | Nymph 1 | — | — | — | — | — | — |
| A5 | 3 | 2024 | *Rhodnius* | Nymph 1 | — | — | — | — | — | — |
| A5 | 4 | 2024 | *Rhodnius* | Nymph 2 | *T. cruzi* | Positive | *T. cruzi* | PX829051 | TcI | — |
| A6 | 1 | 2024 | *Rhodnius* | Adult male | — | — | — | — | — | — |
| A6 | 2 | 2024 | *Rhodnius* | Adult female | — | — | — | — | — | — |
| A7 | 1 | 2024 | *Rhodnius* | Adult female | — | — | — | — | — | — |
| A7 | 2 | 2024 | *Rhodnius* | Adult male | — | — | — | — | — | — |
| A7 | 3 | 2024 | *Rhodnius* | Adult male | *T. cruzi* | Positive | *T. cruzi* | PX829052 | TcI | *Bos taurus* |
| A7 | 4 | 2024 | *Rhodnius* | Nymph 5 | — | — | — | — | — | — |
| A7 | 5 | 2024 | *Rhodnius* | Nymph 5 | *T. cruzi* | Positive | *T. cruzi* | PX829053 | TcI | — |
| A7 | 6 | 2024 | *Rhodnius* | Nymph 5 | *T. cruzi* | Positive | *T. cruzi* | PX829054 | TcI | *Gallus gallus* |
| A7 | 7 | 2024 | *Rhodnius* | Nymph 5 | — | — | — | — | — | — |
| A7 | 8 | 2024 | *Rhodnius* | Nymph 3 | — | — | — | — | — | — |
| A7 | 9 | 2024 | *Rhodnius* | Nymph 2 | — | — | — | — | — | — |
| A7 | 10 | 2024 | *Rhodnius* | Nymph 1 | — | — | — | — | — | — |
| A14 | 1 | 2024 | *Rhodnius* | Adult female | — | Positive | *T. rangeli* | PX829055 | — | — |
| A14 | 2 | 2024 | *Rhodnius* | Nymph 4 | — | Positive | *T. rangeli* | PX829056 | — | — |
| A14 | 3 | 2024 | *Rhodnius* | Nymph 5 | — | Positive | *T. rangeli* | PX829057 | — | — |
| A20 | 1 | 2024 | *Rhodnius* | Adult male | — | Positive | *T. rangeli* | PX829058 | — | *Phylander opossum* |
| A20 | 2 | 2024 | *Rhodnius* | Adult female | *T. cruzi* | Positive | *T. cruzi* | PX829059 | TcI | *Phylander opossum* |
| A20 | 3 | 2024 | *Rhodnius* | Nymph 5 | — | Positive | *T. rangeli* | PX829060 | — | *Phylander opossum* |
| A20 | 4 | 2024 | *Rhodnius* | Nymph 4 | — | — | — | — | — | — |
| A20 | 5 | 2024 | *Rhodnius* | Nymph 4 | — | — | — | — | — | *Phylander opossum* |
| A20 | 6 | 2024 | *Rhodnius* | Nymph 3 | — | Positive | *T. rangeli* | PX829061 | — | *Phylander opossum* |
| A20 | 7 | 2024 | *Rhodnius* | Nymph 3 | — | — | — | — | — | *Copeoglossum nigropunctatum* |
| A20 | 8 | 2024 | *Rhodnius* | Adult male | — | — | — | — | — | — |
| A20 | 9 | 2024 | *Rhodnius* | Nymph 4 | — | Positive | *T. rangeli* | PX829062 | — | *Phylander opossum* |
| A21 | 1 | 2024 | *Rhodnius* | Adult female | — | — | — | — | — | *Hemidactylus mabouia* |
| A21 | 2 | 2024 | *Rhodnius* | Nymph 4 | — | — | — | — | — | *Homo sapiens* |

^a^The table reports specimen-level information, including developmental stage, results of satellite DNA qPCR and 18S rRNA PCR assays, sequencing confirmation and GenBank accession numbers, *T*. *cruzi* DTU, and vertebrate blood meal sources identified by cytb sequencing and BLAST analysis.

^b^A dash (—) indicates no amplification or no data available.
