# Supplementary material for: Deforestation and human proximity influence Trypanosoma cruzi infection in palm-dwelling triatomines
Source: PLoS One. 2026 May 18;21(5):e0349311. doi: 10.1371/journal.pone.0349311 (PMC13183234; doi:10.1371/journal.pone.0349311)
Supplement: Attachment — Submitted filename: Response_to_Reviewers.pdf [file pone.0349311.s005.pdf]

Response to the Editor and Reviewer

Manuscript ID: PONE-D-26-09584

Title: **Deforestation and human proximity influence *Trypanosoma cruzi* infection in palm-dwelling triatomines**

Dear Dr. Saraiva and Reviewers,

We would like to sincerely thank the Academic Editor and the Reviewer for their careful evaluation of our manuscript and for the constructive and valuable comments. We are pleased that the reviewer found the study clearly written, methodologically sound, and scientifically relevant. We have carefully addressed all points raised and revised the manuscript accordingly.

Below we provide a detailed, point-by-point response to all comments.

Response to the Academic Editor's and Journal Requirements

1. Figures 2 and 3 – Copyright and licensing concerns

Comment: Figures may contain copyrighted map or satellite data and may not comply with CC BY 4.0 requirements.

Response:

We thank the editor for raising this important issue. We would like to clarify that Figures 2 and 3 were entirely produced by the authors using non-proprietary and/or original data sources.

Figure 2 was generated using publicly available datasets, including:

- Administrative boundaries from IBGE
- Land use/land cover data from MapBiomas
- Field-collected GPS coordinates

Figure 3 was produced using orthomosaic imagery derived from drone surveys (DJI Mavic 3) conducted by the authors during fieldwork. These images were processed and analyzed entirely by the authors.

Importantly, no proprietary mapping platforms (e.g., Google Maps, Google Earth, or similar services) were used. Therefore, the figures are fully compatible with the CC BY 4.0 license.

In addition, we have revised the figure captions to explicitly state data sources and confirm authorship and originality.

2. ORCID requirement

Response:

We confirm that the ORCID iD of the corresponding author has been validated in the Editorial Manager system.

3. Funding statement inconsistency

Response:

We thank the editor for identifying this issue. We have carefully reviewed the funding information and corrected inconsistencies between the Funding Information section and the Financial Disclosure statement.

4. Funding information in manuscript text

Response:

We confirm that all funding-related information has been removed from the manuscript body and is now included only in the designated Funding Statement section.

5. Ethics statement placement

Response:

We have revised the manuscript to ensure that the ethics statement appears exclusively in the Methods section, as requested. Any duplicate mentions elsewhere in the manuscript have been removed.

6. References formatting

Response:

We have carefully reviewed the reference list and corrected formatting inconsistencies, including font differences and style alignment with PLOS ONE requirements. The revised manuscript now follows the journal's formatting guidelines consistently.

Response to Reviewer #1

We thank Reviewer #1 for the positive evaluation and constructive suggestions.

Comment 1: Inclusion of additional information on *Trypanosoma rangeli* in the Introduction

Response:

We appreciate this insightful suggestion. We have expanded the Introduction to include additional background on *Trypanosoma rangeli*, highlighting:

- Its association with *Rhodnius* spp. triatomines
- Its non-pathogenic nature to humans
- Its epidemiological relevance due to co-circulation with *T. cruzi*
- Its potential to cause cross-reactivity in serological and molecular diagnostic assays, which may lead to false-positive results depending on the diagnostic method used

This addition strengthens the rationale for including *T. rangeli* in our study design.

Comment 2: Figure 2 – add inset map showing Brazil within the Americas

Response:

We thank the reviewer for this helpful suggestion. The inset map indicating the location of Brazil within the Americas was already included in the original Figure 2. However, to improve clarity and visibility, we have revised the figure by enhancing the inset map resolution and improving its visual prominence, as well as better delineating the study area within Brazil.

Comment 3: Reference formatting inconsistency

Response:

We thank the reviewer for identifying this issue. We have corrected the reference formatting to ensure consistency throughout the manuscript according to PLOS ONE style requirements.

We sincerely appreciate the time and effort of the Editor and Reviewer in evaluating our manuscript. We believe that the revisions made have significantly improved the clarity, rigor, and compliance of the manuscript with PLOS ONE requirements.

We hope that the revised version is now suitable for publication in PLOS ONE.

Sincerely,

Gabriel Laporta and co-authors

Gabriel Z. Laporta

Assinado de forma digital por Gabriel Z. Laporta
DN: cn=Gabriel Z. Laporta, o, ou, email=gabriel.laporta@fmabc.br, c=BR
Dados: 2026.04.14 14:53:17 -03'00'
